# All-Atom Four-Body Knowledge-Based Statistical Potentials to Distinguish Native Protein Structures from Nonnative Folds

**DOI:** 10.1155/2017/5760612

**Published:** 2017-10-08

**Authors:** Majid Masso

**Affiliations:** School of Systems Biology, George Mason University, 10900 University Blvd. MS 5B3, Manassas, VA 20110, USA

## Abstract

Recent advances in understanding protein folding have benefitted from coarse-grained representations of protein structures. Empirical energy functions derived from these techniques occasionally succeed in distinguishing native structures from their corresponding ensembles of nonnative folds or decoys which display varying degrees of structural dissimilarity to the native proteins. Here we utilized atomic coordinates of single protein chains, comprising a large diverse training set, to develop and evaluate twelve all-atom four-body statistical potentials obtained by exploring alternative values for a pair of inherent parameters. Delaunay tessellation was performed on the atomic coordinates of each protein to objectively identify all quadruplets of interacting atoms, and atomic potentials were generated via statistical analysis of the data and implementation of the inverted Boltzmann principle. Our potentials were evaluated using benchmarking datasets from Decoys-‘R'-Us, and comparisons were made with twelve other physics- and knowledge-based potentials. Ranking 3rd, our best potential tied CHARMM19 and surpassed AMBER force field potentials. We illustrate how a generalized version of our potential can be used to empirically calculate binding energies for target-ligand complexes, using HIV-1 protease-inhibitor complexes for a practical application. The combined results suggest an accurate and efficient atomic four-body statistical potential for protein structure prediction and assessment.

## 1. Introduction

Over recent years, exponential growth of the Protein Data Bank (PDB) [1] has facilitated the selection of larger, nonredundant subsets of experimentally solved protein structures at higher resolutions, which in turn have provided the data used in developing more effective knowledge-based statistical potentials for improved structure prediction. In contrast to physics-based energy functions, statistical potentials generally perform better and are more computationally efficient at identifying the native structure as a global minimum [2, 3]. Distance-dependent statistical potentials often focus on pairwise atomic contacts within macromolecular structures [4, 5]; however, such energy functions fail to take into consideration important higher-order contributions based on multibody interactions [6, 7]. Indeed, use of an “atomic environment potential” for which neighborhood sizes vary by atom previously demonstrated improved performance at discriminating between native and near-native protein structures [3]. In the present work we employed the well-established computational geometry tiling technique of Delaunay tessellation [8], for objectively identifying all quadruplets of nearest neighbor atoms in order to develop, evaluate, and apply all-atom four-body statistical potentials for protein structure prediction.

**Figure 1:**
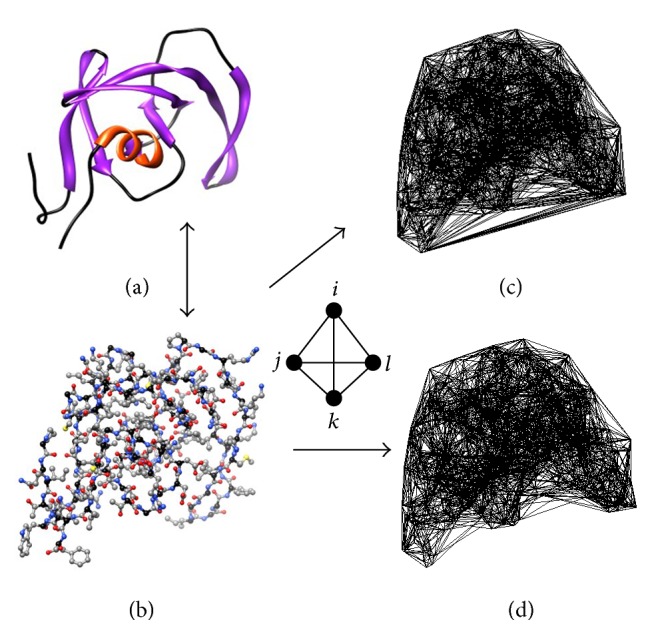
HIV-1 protease (a) ribbon and (b) atomic ball-and-stick diagrams. The atomic coordinates are used as tetrahedral vertices to generate (c) the Delaunay tessellation of the protein chain, a convex hull consisting of thousands of space-filling and nonoverlapping tetrahedra, each of whose vertices objectively identifies a quadruplet of nearest neighbor atoms. The modified tessellation in (d) is obtained by removing all edges longer than 12 Å between pairs of atoms, thereby eliminating all tetrahedra that share those edges and excluding their corresponding atomic quadruplets from consideration as nearest neighbors.

Four-body statistical potentials were derived based on PDB atomic coordinate file data corresponding to single chains selected from over 1400 diverse protein structures. Delaunay tessellation was applied to the three-dimensional (3D) atomic coordinates of each protein chain, whereby atoms were treated as vertices to generate a convex hull encompassing thousands of space-filling, nonoverlapping, irregular tetrahedra (Figure 1). For assurance that each tetrahedron identifies at its four vertices a quadruplet of atoms that are pairwise all within a prescribed distance from one another, a subsequent edge-length cutoff parameter may be introduced; removal of a tetrahedral edge between a pair of atoms longer than this cutoff eliminates from the tessellation all tetrahedra sharing that edge. Depending on the size *K* of the atomic alphabet used for labeling points, the four atoms appearing at vertices of any particular tetrahedron in these tessellations represent one of 35 (*K* = 4 letters), 330 (*K* = 8), or 8855 (*K* = 20) possible distinct atomic quadruplet types. For each cutoff (if any) and alphabet size, statistical data obtained from the protein chains and their tessellations included the following: (1) observed relative frequencies of interaction for each type of atomic quadruplet, based on their rates of occurrence as tetrahedral vertices; and (2) rates expected by chance for each atomic quadruplet type, based on relative frequencies of individual atom types in the protein chains and use of a multinomial reference distribution. Through application of the inverted Boltzmann principle [9, 10], the negative logarithm of the ratio of observed to expected rates of occurrence was used to calculate an empirical energy of interaction for each atomic quadruplet, which collectively form an atomic four-body statistical potential.

The approach implemented here at the atomic level was motivated by its prior successful application at the residue level [11–16]. All atomic 3D coordinates in proteins are considered in this work to generate the all-atom four-body statistical potentials, while previously developed residue-based four-body potentials used only a single point per amino acid (e.g., C_*α*_ or residue center of mass). Clearly, there is a degree of information loss with the coarser-grained residue representation of proteins relative to the finer all-atom representation. Both approaches implement the Delaunay tessellation algorithm, which uses the respective point-sets to serve as vertices for generating a tetrahedral tiling of the protein structure that objectively identifies quadruplets of nearest neighbors (i.e., either residues or atoms). Given its significantly more sparse point-set, a residue-based (i.e., one point per residue) tessellation typically yields a few hundred tetrahedra, whereas tessellation applied to all the atoms in the same protein structure has on the order of a few thousand tetrahedra.

Upon selecting an atomic alphabet and edge-length cutoff as parameters, the energy of any folded protein chain would subsequently be calculated with the atomic four-body potential as follows: label and tessellate the 3D atomic coordinates of the structure according to the same parameters, refer to the previously derived atomic four-body potential under those parameters to assign a score to each tetrahedron in the tessellation equal to the interaction energy of the atomic quadruplet found at its four vertices, and add up the scores of all the tetrahedra in the tessellation. The all-atom four-body statistical potentials that we developed were each evaluated by scoring multiple decoy directories in the Decoys-‘R'-Us benchmarking database [17]. We compared these four-body potentials to one another, based on standard performance metrics, as well as to the knowledge-based potentials of Fogolari et al. [18] and Summa et al. [3]; the latter study detailed performance results for 10 diverse physics- and knowledge-based potentials to conduct their own comparisons, hence providing us an opportunity to assess our four-body potentials relative to a dozen other methods in total. Lastly, we report on a practical application, related to predicting target-inhibitor binding energy, by implementing a modification of our best performing four-body potential.

## 2. Methods

### 2.1. Protein Training Set

A nonredundant set of 1417 high-resolution (≤2.2 Å) crystallographic structures, with atomic coordinate files deposited in the PDB, were culled using the PISCES server [28] with the constraint that the single protein chains selected from the structures shared low (<30%) sequence identity (http://binf2.gmu.edu/automute/tessellatable1417.txt). The ensemble of structure files is diverse, consisting of single- and multichain proteins, the vast majority of which are additionally complexed to small molecular or peptide ligands. Coordinates of hydrogen atoms and water molecules were removed from all files prior to proceeding with the analyses.

### 2.2. Designation of Atoms

For each of the 1417 protein chains, three alphabets were explored for defining atom types and labeling points corresponding to their 3D atomic coordinates. In the first instance, a simple four-letter alphabet (C, N, O, and S) accounts for all atoms and ensures sufficient frequency data are collected for all possible atomic quadruplets observed at the four vertices of tetrahedra in Delaunay tessellations (Table 1). Clearly, the same atom type may appear at more than one of the four vertices of any tetrahedron in a protein tessellation, and given that those vertices are unordered, all permutations of the four atomic letters at the vertices of a tetrahedron refer to the same quadruplet, so that an alphabetical ordering (e.g., COON) of the atoms can be used as a singular representation. In this case, a combinatorial argument [29] shows that the number *N* of distinct subsets of size *r* = 4 letters that can be formed from an atomic alphabet of size *K* is given by

(1) $$\begin{array}{c} N=\left(\begin{array}{c} K+r-1\\ r \end{array}\right)=\left(\begin{array}{c} K+3\\ 4 \end{array}\right). \end{array}$$

Hence, *K* = 4 letters admit *N* = 35 distinct atomic quadruplets. Next, an atomic alphabet consisting of *K* = 8 letters (amino acid backbone: N_B_, C_*α*_, C_B_, and O_B_; side-chain: N_S_, C_S_, O_S_, and S) differentiates between backbone alpha- and carbonyl-carbon atoms, distinguishes residue backbone atoms from those in side-chains, and can form *N* = 330 distinct atomic quadruplets. Lastly, we explored a maximum diversity of quadruplet atomic interactions with *K* = 20 letters as described in Summa et al. [3], which groups atoms based on common traits, including bonding pattern, partial charge, and hydrophobicity, and generates *N* = 8855 distinct atomic quadruplets.

### 2.3. Derivation of the Atomic Four-Body Statistical Potentials

Delaunay tessellations for the 1417 single protein chains were generated by submitting their respective atomic coordinates as input to the Qhull program [30], visualizations of the tessellated structures were obtained by utilizing the output data from Qhull to create plots within Matlab, and molecular graphics were produced with Chimera [31] (Figure 1). An in-house suite of Perl programs was used for all data formatting and analyses related to the tessellated structures (Table 1). In particular, for each atomic alphabet of size *K* the relative frequencies of occurrence *f*_*ijkl*_ for all *N* types of atomic quadruplets (*i*, *j*, *k*, *l*) were calculated as the proportion of tetrahedra among all the tessellations for which the four atoms appear on the vertices. Four separate sets of relative frequencies were calculated for each of the three atomic alphabets explored, based on the original protein tessellations (no edge-length cutoff applied), as well as tessellations modified by introducing cutoffs of length 12 Å, 8 Å, and 4.8 Å. The use of an 8 Å cutoff is consistent with that used by other researchers to generate atomic pair potentials [32], while the other two cutoffs were also selected to identify the appropriate choice for an atomic four-body potential.

For each of the three atomic alphabets, we additionally computed relative frequencies of occurrence *a*_*n*_, *n* = 1,…, *K* for the *K* atom types in all 1417 single protein chains. These frequencies, in turn, were needed for calculating the rate *p*_*ijkl*_ expected by chance for all *N* types of atomic quadruplets (*i*, *j*, *k*, *l*) obtained using a multinomial reference distribution, given by

(2) pijkl=4!∏n=1Ktn!∏n=1Kantn,where∑n=1Kan=1,  ∑n=1Ktn=4.

**Figure 2:**
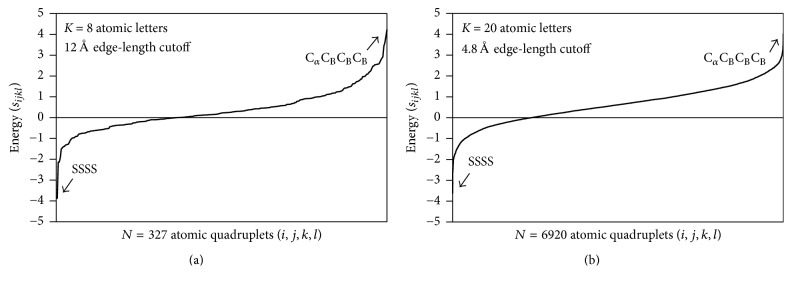
Graphical representations for two four-body potentials, based on an eight-letter alphabet with a 12 Å edge-length cutoff, and a twenty-letter alphabet with a 4.8 Å edge-length cutoff. Here C_*α*_ = alpha-carbon, C_B_ = backbone carbonyl-carbon, and S = side-chain sulfur (from either cysteine or methionine) represent the same atom types in both alphabets, with quadruplets SSSS and C_*α*_C_B_C_B_C_B_ appearing at the same extremes of both potentials. Despite millions of tetrahedra generated by the 1417 protein tessellations irrespective of the cutoff length (see Table 1), note that 3 of 330 atomic quadruplet types (C_*α*_C_*α*_C_*α*_S, C_B_C_B_C_B_C_B_, and C_*α*_C_B_C_B_N_S_) did not appear at all as tetrahedral vertices based on an 8-letter atomic alphabet with a 12 Å cutoff (N_S_ = side-chain nitrogen atom), while 1935 of 8855 quadruplets types were not observed under a 20-letter alphabet with a 4.8 Å cutoff.

In the formula above, *t*_*n*_ represents the number of occurrences of atom type *n* in the quadruplet. For each pair of parameters selected (i.e., alphabet size and cutoff), we applied the inverted Boltzmann principle to calculate a score *s*_*ijkl*_ = –log⁡(*f*_*ijkl*_/*p*_*ijkl*_) for quantifying the interaction energy for all *N* types of atomic quadruplets (*i*, *j*, *k*, *l*), as described by Sippl [9, 10], thus defining a particular atomic four-body statistical potential function (Table 2 and Figure 2). A total of 12 four-body potentials were generated and evaluated in this study (3 atomic alphabets × 4 edge-length cutoffs, which includes the case where no cutoff is applied to the original tessellations).

In any atomic tessellation of a protein structure, two adjacent tetrahedra may share a common vertex (1 atom), a common edge (2 atoms), or a common triangular face (3 atoms). Although the two adjacent tetrahedra represent two sets of atomic quadruplets that may share up to 3 atoms in common, those quadruplets are distinct by virtue of the atom(s) that the two tetrahedra do not share. The collective interaction of a quadruplet of atoms as a fundamental unit in this four-body scenario is analogous to the interaction of two atoms in the development of a pair potential, whereby a given atom may be considered to interact with each of several neighboring atoms by virtue of satisfying a prescribed distance cutoff between itself and each of the neighbors, and therefore the atom is shared by all of those pairs; likewise, two atomic quadruplets from adjacent tetrahedra in a tessellation may share up to 3 atoms and yet remain fundamentally distinct quadruplets. Moreover, since Delaunay tessellation does not distinguish types of bonds and generates a tetrahedral tiling by objectively identifying quadruplets of nearest neighbor atoms based solely on their six collective pairwise distances from each other, all covalent bonds as well as noncovalent interactions between particular pairs of atoms are included together in these tetrahedral atomic quadruplets without the need to explicitly identify and segregate them. Recent studies suggest that covalent interactions are informative when combined with nonbonded interactions [33, 34].

### 2.4. Decoy Database

A significant collection of models provided in the Decoys-‘R'-Us database (http://compbio.buffalo.edu/dd/) form a well-established and challenging standard for benchmarking the performance of energy functions. Several categories are located under the heading “The multiple decoy sets,” each containing a number of decoy model directories. Each such directory is named after the PDB accession code of the native crystallographic protein structure and contains coordinate files for that native structure as well as for numerous decoy model structures (i.e., alternative conformations for a given native structure); additionally, the directory includes a file that provides the C_*α*_ root mean square deviations (rmsds) for all the alternative models relative to the native structure. For this work, we focused on the following decoy set categories: 4_state_reduced, fisa, fisa_casp3, hg_structal, ig_structal, ig_structal_hires, lattice_ssfit, and lmds.

## 3. Results

### 3.1. Energy Calculations and Benchmark Evaluation Measures

Energy calculations were made for 145 native protein structures as well as for all of their respective decoy models downloaded from the 8 decoy set categories in the Decoys-‘R'-Us database. To this end, all native and decoy structures were tessellated, and their energies were repeatedly computed using all twelve four-body potentials under their respective parameters of atomic alphabet size and tessellation edge-length cutoff. Given the energy scores for a native structure and its collection of decoys, all calculated using the same four-body potential, the following measures of performance were evaluated.

*(1) Native Rank*. Among the native protein and all decoys, the structures are ranked in ascending order according to increasing energy (i.e., lowest energy structure has rank 1).

*(2) Z-Score*. This measurement is defined as

(3) $$\begin{array}{c} z=-\frac{E_{n}-\langle E\rangle }{\sigma }, \end{array}$$

where *E*_*n*_ is energy of the native structure, 〈*E*〉 is mean energy over all decoy models, and *σ* is standard deviation of the distribution of decoy energies [3]. A large positive *z*-score indicates a wide gap between the energy of the native protein and the mean decoy energy.

*(3) Correlation Coefficient (r)*. It is the linear correlation between calculated energy and rmsd. For decoys with low rmsds relative to the native structure, good correlation is preferable; however, this is unlikely if decoys are significantly misfolded with high rmsds.

*(4) Fractional Enrichment (FE)*. It is the proportion of decoy structures corresponding to the lowest 10% of rmsds that are also found among those corresponding to the lowest 10% of calculated energy scores.

The raw performance data obtained with the four-body potential derived using a 4-letter atomic alphabet and a 12 Å tessellation edge-length cutoff as parameters (Table 2) are presented in Table 3. As such, we employed the same 4-letter alphabet to label the atomic vertices in the tessellations of the 145 native structures and all of their respective decoys, and edges longer than 12 Å were removed from all tessellations prior to calculating total energies as described in the last paragraph of the Introduction. Data analogous to that of Table 3 were obtained using each of the 11 other four-body potentials generated for this study under alternative parameter-pair values for atomic alphabet size and tessellation edge-length cutoff (raw data not shown).

**Figure 3:**
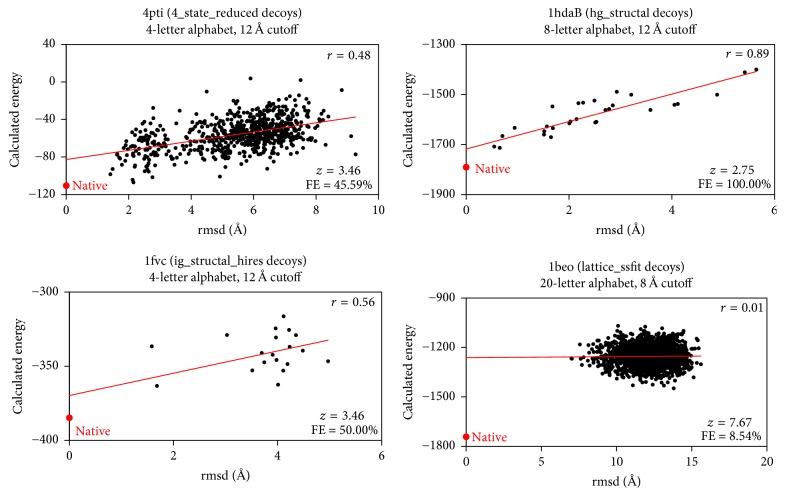
Sampling of calculated energy versus rmsd plots for four decoy sets. A different atomic four-body statistical potential energy function (i.e., distinct pairs of atomic alphabet size and tessellation edge-length cutoff parameters) was selected to compute the energy values for each plot. The plots reveal wide variability in the number of alternative conformations for a given native structure based on decoy category, and they highlight the relative strengths and weaknesses of native rank, correlation coefficient (*r*), *z*-score, and fractional enrichment (FE) as performance measures under a range of conditions, hence reinforcing their collective importance for evaluating energy functions.

The plots of energy versus rmsd in Figure 3, based on 4 native proteins and their collections of decoys evaluated with a varied selection of four-body potentials that we generated, are illustrative of the strengths and weaknesses of the performance measures defined above. In particular, since 4state_reduced is known to contain native-like alternative conformations for each protein in the set, reasonably good correlation (*r* ≫ 0) and fractional enrichment (FE > 10%) are expected from a reliable energy function [18, 35], and this is illustrated by the plot for 4pti. Next, ig_structal_hires and hg-structal contain decoys built by homology modeling for immunoglobulin (ig) and globin (hg) proteins, all of which are native-like structures with very low rmsds relative to native [18]. The plots for 1fvc and 1hdaB reflect the expected strong correlation and fractional enrichment; additionally, despite the fact that the native protein and very low rmsd decoys all have a good chance of achieving the lowest energy conformation, both native proteins rank 1 for these examples. Finally, the set lattice_ssfit consists of decoys selected with an all-atom energy function and refined using coarse lattice models, and rmsd > 4 Å for all decoys in this set relative to their native proteins [18, 36]. The plot for 1beo shows that, as expected in such cases of significantly misfolded decoys, there is no correlation between energy and rmsd relative to the native structure; furthermore, the fractional enrichment is low and the *z*-score is relatively large, as commonly encountered by such decoys and suggested by the plot.

### 3.2. Four-Body Potentials: Relative Performance

To effectively rank all twelve four-body potentials generated for this study, first we identified, for each of the 145 native proteins and their respective decoys, the best native rank and largest *z*-score, correlation coefficient, and fractional enrichment values obtained, without regard to which potential yielded those optimal values of the performance measures. Next, for each potential separately, we counted the number of times (out of 145) that the potential either matched or singularly provided each optimal value recorded for a performance measure concerning a native protein and its set of decoys (Table 4, numbers above parentheses). For each performance measure, we then ranked these counts across all the potentials (Table 4, numbers in parentheses); subsequently for each potential separately, we averaged its rankings across the four performance measures (Table 4, next to bottom row). Finally, those averaged ranks were used to generate an overall ranking of the twelve four-body potentials (Table 4, bottom row). The ranking approach based on relative performance employed here and in the subsequent section was inspired by the technique described in Summa et al. [3] for comparing the performance of their potential to other related methods.

In general, four-body potentials derived using a 4-letter atomic alphabet ranked highest, followed by those based on *K* = 20 letters, while potentials generated using 8 atom types ranked poorly over all four choices of tessellation edge-length cutoff parameter values. Among the 4-letter alphabet potentials, the one based on full structure tessellations (i.e., no edge-length cutoff) outperformed that using a 12 Å cutoff; however, the latter case is preferable since, without a fixed cutoff, false-positive atomic quadruplet interactions are admitted into the analyses based on those tessellations. A satisfying solution to this dilemma is revealed in the subsequent section as these four-body potentials are compared to those developed by other research groups.

### 3.3. Relative Performance: Comparisons with Related Methods

Next, an approach similar to that described in the previous section is used to individually compare each of our 12 four-body potentials to those of a dozen related methods. Using the Decoys-‘R'-Us database, Summa et al. [3] compared their “atomic environment potential” to ten other well-known physics- and knowledge-based potentials, providing us with valuable raw data to make comparisons with our four-body potentials. In addition, Fogolari et al. [18] developed an energy function employing two centers of interactions per amino acid. They also used the Decoys-‘R'-Us database to evaluate its performance, and these data are also included in our evaluations. Out of the 145 decoy sets that we used for benchmarking our four-body potentials relative to one another, 129 sets overlap with those used by both of those studies and form the basis of comparisons reported here. Lastly, the ten related methods investigated by Summa et al. [3] for comparing relative performance and used by us for a similar purpose include the following: three taken from the AMBER force field (a simple van der Waals potential, a pairwise electrostatic potential term, and the sum of these two terms, the latter representing the entire nonbonded contact energy of a typical molecular mechanics force field without either an explicit or implicit solvent model) [19]; two taken from CHARMM19 (both a van der Waals and a coulombic term) [20]; the Δ*E* and Δ*E*^solv^ potentials of Delarue and Koehl [21], and the Δ*G*^env^ potential of Koehl and Delarue [22]; and distance-dependent atomic potentials RAPDF [23] and DFIRE [24].

For each of the 129 decoy sets common to the studies, we obtained the raw performance data (i.e., native rank, *z*-score, correlation, coefficient, and fractional enrichment as presented in Table 3 for one of the four-body potentials) generated by each of the twelve methods described above. Next, we selected one of our four-body potentials and included its raw performance data, for a total of 13 methods to be compared. With every decoy set, we identified the best native rank achieved and the largest values obtained for *z*-score, correlation coefficient, and fractional enrichment, without regard to which of the 13 methods was responsible for each optimal measurement. For each of these 13 methods separately, we counted the number of times (out of 129) that the method either matched or singularly provided each optimal value recorded for a performance measure concerning a native protein and its set of decoys (Table 5, numbers to the left of those in parentheses). For each performance measure, we then ranked these counts across all 13 methods (Table 5, numbers in parentheses); subsequently for each method separately, we averaged its rankings across the four performance measures (Table 5, next to last column). Finally, those averaged ranks were used to generate an overall ranking of the 13 methods (Table 5, last column).

The overall rankings in Table 5 reveal that our four-body potential, derived using a 4-letter alphabet and 12 Å cutoff as parameters, outperformed 9 other methods, tied in overall ranking (3rd) with the coulombic term from CHARMM19, and was outperformed by DFIRE and the “atomic environment potential” of Summa et al. The methodology described in the previous paragraph was repeated separately for each of the twelve four-body potentials that we investigated, and the overall rankings in each case are reported in the columns of Table 6. Four-body potentials employing a 4-letter alphabet again appear to be the most competitive, and in particular those derived using unmodified tessellations (i.e., no edge-length cutoff) and a 12 Å cutoff achieved the highest overall rankings (3rd) among all of the four-body potentials, each in comparison to the 12 related state-of-the-art methods. As mentioned in the prior section, the introduction of false-positive atomic quadruplet interactions into the analyses is a concern when edge-length cutoffs are not considered after tessellation. Given that both of these potentials are equally competitive when compared with the other related methods, we conclude that a 4-letter alphabet and 12 Å tessellation edge-length cutoff provide the best pair of parameters with which to derive a four-body potential for calculating protein structure energies and effectively distinguishing native folds from nonnative decoy structures.

## 4. Discussion

Energy calculations for single protein chains have been the sole focus up to this point, so it has been appropriate to consider atomic alphabets based only on the four heavy atom types found in proteins: carbon, nitrogen, oxygen, and sulfur. As mentioned earlier in Methods, a training set of 1417 diverse structures of single chain proteins were used for deriving the four-body potentials; however, the atomic coordinate for these protein chains was each obtained from a distinct PDB coordinate file, and the vast majority of these files are for structures of proteins complexed to small molecular or peptide ligands. Therefore, in order to tessellate the entirety of each of these PDB files, an expansion of the atomic alphabet is necessary to accommodate all atom types. The fact that such tessellations have an important function will become apparent as an application is introduced for predicting target-ligand binding affinities.

### 4.1. Generalized Four-Body Potential: An Alphabet Incorporating All Atom Types

Given the impressive performance on protein structures by the four-body potential derived using a 4-letter atomic alphabet and 12 Å tessellation edge-length cutoff, we simply expanded the alphabet to 6 letters in order to include atoms found exclusively in molecular ligands: M = all metals and X = all nonmetals other than (N, C, O, S). The atomic frequency data and total number of tetrahedra generated by tessellating the totality of the atomic coordinate data in the 1417 PDB structure files (hydrogen atoms and water molecules excluded, as discussed in Methods), after filtering out edges longer than 12 Å, are provided in Table 7. Since we are now working with a *K* = 6 letter alphabet, the atoms at the four vertices of each tetrahedron of a tessellation represent one of *N* = 126 atomic quadruplets. A retracing of the steps described in Methods yields the all-atom four-body statistical potential presented in Table 8. Note that 11 of the 126 atomic quadruplet types are not represented by any of the 36,406,467 tetrahedra obtained from the tessellations.

### 4.2. Application: Target-Ligand Binding Affinity Prediction

**Figure 4:**
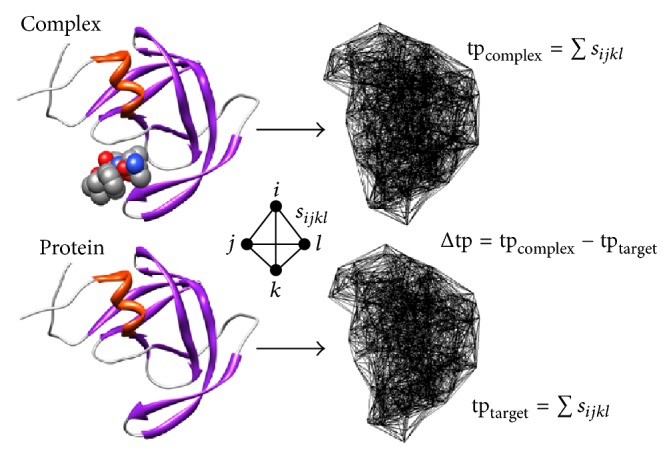
Visualization of a procedure based on a simplified model to calculate target-ligand binding affinity (Δtp) with the four-body potential.

The four-body potential derived in the previous section can be used to calculate the energy of any macromolecular structure. First, the 3D atomic coordinates of the structure are each labeled using the 6-letter alphabet and those points are tessellated subject to a 12 Å cutoff, then each tetrahedron in the tessellation is scored according to the atomic quadruplet identified at its four vertices by referring to the four-body potential previously derived in Table 8, and finally, the scores of all the tetrahedra are added up to determine the energy of the structure. Using the notation tp (i.e., total potential) to refer to the energy of a structure calculated in this way, we empirically calculate target-ligand binding affinity in the following manner (Figure 4):

(1) Tessellate the entire macromolecular complex and calculate tp_complex_.
(2) Tessellate only atomic coordinates for the target protein and calculate tp_target_.
(3) The calculated target-ligand binding affinity is given by the difference
  (4) $$\begin{array}{c} \Delta tp=tp_{complex}-tp_{protein}. \end{array}$$

The above formula is a simplified model that is valid in the case of small ligands for which tetrahedra formed at the protein interface dominate any purely internal quadruplet atomic interactions within the ligand; hence, the relative energy contribution of the ligand is negligible [37].

### 4.3. Example: Predicting HIV-1 Protease-Inhibitor Binding Energy

**Figure 5:**
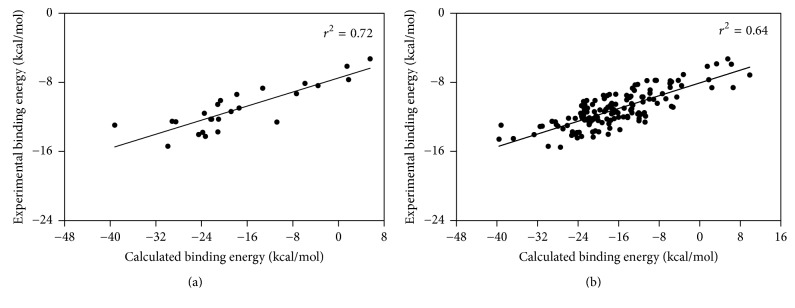
Scatter plots of experimental versus calculated binding energy for (a) twenty-five HIV-1 protease-inhibitor complexes culled by Jenwitheesuk and Samudrala [25] and (b) a larger set of 140 such complexes which include the initial twenty-five, with the remainder obtained by searching the Binding MOAD database [26, 27]. Both experimental binding energies and crystallographic structures are available for these complexes, and the latter were required for calculating binding energy (Δ*ts*) as outlined in the text and in Figure 4.

To validate the approach for empirically calculating binding affinity, PDB accession codes and experimental binding energies were obtained from Jenwitheesuk and Samudrala [25] for twenty-five HIV-1 protease-inhibitor complexes (Table 9); they converted experimental inhibition constants (*K*_*i*_) to experimental binding energies (Δ*G*^0^, Gibbs free energy of binding, in units of kcal/mol) by applying the equation Δ*G*^0^ = −*RT*ln⁡(*K*_*i*_), where *R* is the gas constant (1.987 cal K^−1^ mol^−1^) and *T* is the absolute temperature (room temperature, 300 K). By following the steps outlined in the previous section, we determined Δtp for each of these complexes and used it as the calculated binding energy. As shown in Figure 5, the experimental and calculated binding energies for these complexes were highly correlated (*r*^2^ = 0.72). From a subsequent search of the Binding MOAD database [26, 27], we identified 115 additional HIV-1 protease-inhibitor complexes with experimental structures in PDB, for which experimental inhibition constants are available (Table 9). As before, Δtp values were obtained and used for representing the calculated binding energy, and *K*_*i*_ values were converted to experimental binding energies. The correlation remained robust (*r*^2^ = 0.64) when these data were combined with those of the initial plot (Figure 5).

## 5. Conclusion

In this study, we derived and evaluated twelve distinct atomic four-body knowledge-based statistical potentials for protein structure prediction, by altering two parameter values: atom type (4-, 8-, or 20-letter alphabets) and distance cutoff for atomic interactions (none, 12 Å, 8 Å, or 4.8 Å). The best potential employed a simple 4-letter atomic alphabet and considered any quadruplet of atoms to be interacting when they were all pairwise within 12 Å of each other. In a head-to-head comparison of methods using 129 benchmarks from the Decoys-‘R'-Us database, our potential ranked 3rd and was outperformed by only two out of twelve other state-of-the-art methods. In addition to its simplicity and relative accuracy, our method is faster and more efficient in general, with some of the other physics- and knowledge-based potentials used for comparison employing well over one hundred different atom types. Future plans for improvement include combining this four-body potential together with other knowledge-based potentials, as well as subsequently implementing them together in conjunction with statistical machine learning tools.

## Figures and Tables

**Table 1:** Summary data for the protein structure training set (1417 single chains).

Atomic alphabet	Count	Proportion
C	1572222	0.634149
N	425874	0.171774
O	469869	0.189520
S	11299	0.004557

^*∗*^Total atom count	2479264	

^*∗*^Total tetrahedron counts		
No edge-length cutoff	16152638	
12 Å edge-length cutoff	15497203	
8 Å edge-length cutoff	14567713	
4.8 Å edge-length cutoff	9569503	

^*∗*^Same counts regardless of atomic alphabet size.

**Table 2:** Atomic four-body statistical potentials employing a 4-letter alphabet.

Quad	No cutoff		12 Å cutoff		8 Å cutoff		4.8 Å cutoff	
	Count	*s*_*ijkl*_	Count	*s*_*ijkl*_	Count	*s*_*ijkl*_	Count	*s*_*ijkl*_
CCCC	1711740	0.183570	1692278	0.170562	1643281	0.156444	922742	0.224573
CCCN	1823746	0.190871	1790819	0.180796	1726843	0.169730	1229054	0.134910
CCCO	2807489	0.046213	2750767	0.037094	2616669	0.031930	1533444	0.081509
CCCS	119435	−0.201538	117868	−0.213776	114416	−0.227745	53175	−0.077468
CCNN	832442	0.140340	803373	0.137776	764343	0.132553	612799	0.046021
CCNO	3838549	−0.179748	3746112	−0.187158	3593186	−0.195913	2695736	−0.253612
CCNS	53655	0.055872	52228	0.049592	50119	0.040628	28817	0.098480
CCOO	1643096	−0.069578	1559797	−0.064976	1408171	−0.047423	796121	0.017752
CCOS	86638	−0.109530	84188	−0.115055	79331	−0.116117	36077	0.043594
CCSS	6408	−0.898511	6343	−0.912057	6183	−0.927840	3685	−0.885580
CNNN	64504	0.507783	51089	0.591028	39927	0.671251	18568	0.821250
CNNO	961282	−0.145664	903031	−0.136526	842880	−0.133431	647785	−0.201598
CNNS	7628	0.335839	6916	0.360394	6293	0.374540	3499	0.446952
CNOO	1380693	−0.260215	1283449	−0.246502	1168615	−0.232641	757532	−0.226873
CNOS	44097	−0.082434	41962	−0.078877	39453	−0.078957	22706	−0.021519
CNSS	2153	−0.691035	2131	−0.704562	2085	−0.721950	1312	−0.703279
COOO	336824	−0.081946	278989	−0.018132	207773	0.083015	65646	0.400894
COOS	17883	0.051201	16090	0.079093	13492	0.128713	4711	0.403174
COSS	2068	−0.630846	2009	−0.636259	1897	−0.638215	1001	−0.543084
CSSS	214	−1.741768	214	−1.759742	207	−1.772176	125	−1.735618
NNNN	4632	0.482308	2068	0.814499	1118	1.054783	133	1.796871
NNNO	36223	0.233848	22370	0.425144	15881	0.547103	5948	0.791107
NNNS	407	0.564301	263	0.735926	203	0.821548	58	1.183114
NNOO	190771	−0.268893	158110	−0.205359	137906	−0.172816	97822	−0.206171
NNOS	3088	0.204035	2530	0.272582	2171	0.312201	1061	0.440643
NNSS	236	−0.599166	230	−0.605982	224	−0.621354	79	−0.351235
NOOO	129494	−0.234026	92243	−0.104725	67495	0.004100	26079	0.234579
NOOS	5426	0.001929	4626	0.053197	4007	0.088737	1832	0.246129
NOSS	436	−0.522015	418	−0.521701	405	−0.534836	220	−0.452305
NSSS	138	−2.118466	138	−2.136453	137	−2.160159	48	−1.887182
OOOO	39551	−0.278298	23332	−0.067103	12170	0.188717	1542	0.903421
OOOS	1462	0.137035	1007	0.280952	636	0.453674	78	1.182534
OOSS	158	−0.339520	143	−0.314191	125	−0.282625	32	0.126633
OSSS	22	−1.278314	22	−1.296297	21	−1.302962	5	−0.862215
SSSS	50	−3.855858	50	−3.873832	50	−3.900710	31	−3.875603

**Table 3:** Performance evaluation on 145 benchmarks in 8 decoy sets from Decoys-‘R'-Us, based on energies obtained with the four-body potential derived using a 4-letter alphabet and 12 Å cutoff as parameters.

Decoy set	PDB ID	^*∗*^Native rank	*z*-score	^*∗*^ *r*	^*∗*^FE
4state_reduced	1ctf	156/631	0.7	0.27	11.1
	1r69	6/676	2.5	0.18	14.9
	1sn3	23/660	1.8	0.42	48.5
	2cro	5/674	2.4	0.40	20.9
	3icb	137/654	0.8	0.34	21.5
	4pti	1/687	3.5	0.48	45.6
	4rxn	83/677	1.1	0.31	19.4

fisa	1fc2	497/501	−2.2	−0.22	4.0
	1hddC	442/501	−1.2	−0.02	8.0
	2cro	32/501	1.5	0.07	10.0
	4icb	465/500	−1.4	−0.02	6.0

fisa_casp3	1bg8A	10/1201	2.4	0.06	14.2
	1bl0	883/972	−1.4	−0.19	5.2
	1eh2	1624/2414	−0.5	0.09	16.2
	1jwe	539/1408	0.3	0.04	9.3
	smd3	1/1201	2.8	0.07	10.0

hg_structal	1ash	2/30	2.2	0.47	33.3
	1babB	1/30	3.7	0.17	66.7
	1colA	1/30	3.9	0.46	33.3
	1cpcA	4/30	1.4	0.19	0.0
	1ecd	11/30	0.3	0.00	0.0
	1emy	1/30	3.3	0.38	66.7
	1flp	1/30	2.3	0.21	66.7
	1gdm	3/30	1.5	0.12	33.3
	1hbg	1/30	2.8	0.03	33.3
	1hbhA	1/30	3.4	0.18	33.3
	1hbhB	3/30	1.7	−0.27	33.3
	1hdaA	1/30	5.2	0.27	33.3
	1hdaB	1/30	4.2	0.42	33.3
	1hlb	1/30	2.0	0.25	33.3
	1hlm	5/30	0.9	0.12	0.0
	1hsy	12/30	0.3	0.49	33.3
	1ithA	1/30	3.4	0.17	66.7
	1lht	13/30	0.1	0.12	0.0
	1mba	1/30	5.7	0.31	33.3
	1mbs	30/30	−2.8	0.16	33.3
	1mygA	3/30	1.3	0.25	66.7
	1myjA	14/30	0.1	0.29	0.0
	1myt	20/30	−0.8	0.06	33.3
	2dhbA	30/30	−2.0	−0.23	33.3
	2dhbB	24/30	−0.9	0.45	33.3
	2lhb	1/30	4.7	0.49	66.7
	2pghA	6/30	1.1	0.24	33.3
	2pghB	1/30	2.7	0.29	33.3
	4sdhA	1/30	2.7	−0.01	33.3

lattice_ssfit	1beo	1/1999	4.9	0.04	7.0
	1ctf	88/2001	1.7	−0.04	11.0
	1dktA	13/1999	2.5	0.00	8.5
	1fca	3/2001	3.1	0.03	16.0
	1nkl	92/1998	1.7	0.01	17.1
	1pgb	506/2000	0.7	−0.04	13.0
	1trlA	68/2000	1.8	0.03	10.5
	4icb	223/2000	1.2	−0.03	13.0

ig_structal_hires	1dvf	1/20	2.1	0.13	50.0
	1fgv	1/20	3.9	0.20	50.0
	1flr	16/20	−0.3	−0.08	0.0
	1fvc	1/20	3.5	0.56	50.0
	1gaf	1/20	3.8	−0.01	50.0
	1hil	1/20	2.6	0.55	50.0
	1ind	7/20	0.2	0.36	0.0
	1kem	1/20	2.4	0.46	50.0
	1mfa	1/20	2.0	0.10	50.0
	1mlb	9/20	0.2	−0.51	0.0
	1nbv	11/20	−0.2	0.20	0.0
	1opg	20/20	−3.8	−0.45	0.0
	1vfa	1/20	2.2	0.39	50.0
	1vge	2/20	1.9	0.42	50.0
	2cgr	1/20	2.6	0.39	50.0
	2fb4	7/20	0.3	−0.09	0.0
	2fbj	19/20	−1.1	−0.12	0.0
	6fab	3/20	1.5	0.25	0.0
	7fab	10/20	0.2	0.27	0.0
	8fab	1/20	2.6	0.14	50.0

ig_structal	1acy	56/61	−1.5	−0.01	0.0
	1baf	3/61	2.1	0.12	16.7
	1bbd	61/61	−3.1	−0.15	0.0
	1bbj	23/61	0.1	−0.02	0.0
	1dbb	24/61	0.1	−0.31	0.0
	1dfb	8/61	1.1	0.20	0.0
	1dvf	1/61	2.7	0.11	16.7
	1eap	1/61	2.6	0.16	16.7
	1fai	2/61	1.9	0.29	33.3
	1fbi	12/61	0.8	−0.06	33.3
	1fgv	1/61	2.9	0.03	16.7
	1fig	2/61	2.0	0.11	16.7
	1flr	40/61	−0.2	−0.08	16.7
	1for	1/61	3.5	0.16	33.3
	1fpt	1/61	3.7	0.12	16.7
	1frg	4/61	1.5	0.39	16.7
	1fvc	1/61	2.8	0.16	33.3
	1fvd	9/61	1.1	0.09	33.3
	1gaf	1/61	3.5	0.04	16.7
	1ggi	9/61	1.1	−0.03	0.0
	1gig	10/61	1.0	0.16	0.0
	1hil	2/61	2.5	0.41	16.7
	1hkl	1/61	4.3	0.01	16.7
	1iai	1/61	3.0	0.15	33.3
	1ibg	30/61	0.0	−0.08	16.7
	1igc	55/61	−1.3	0.02	0.0
	1igf	6/61	1.3	0.10	16.7
	1igi	2/61	1.9	0.13	16.7
	1igm	43/61	−0.5	0.09	0.0
	1ikf	1/61	2.7	0.35	50.0
	1ind	20/61	0.5	0.31	0.0
	1jel	1/61	3.5	0.02	16.7
	1jhl	1/61	3.1	0.16	33.3
	1kem	1/61	2.8	0.26	33.3
	1mam	1/61	2.0	0.31	33.3
	1mcp	60/61	−1.8	0.23	0.0
	1mfa	4/61	1.6	−0.01	16.7
	1mlb	23/61	0.4	−0.19	0.0
	1mrd	2/61	2.6	0.28	33.3
	1nbv	36/61	−0.2	0.03	0.0
	1ncb	12/61	0.9	−0.02	16.7
	1ngq	13/61	0.8	0.00	0.0
	1nmb	1/61	2.9	0.38	16.7
	1nsn	8/61	1.0	−0.11	0.0
	1opg	61/61	−2.7	−0.01	0.0
	1plg	2/61	1.8	−0.02	16.7
	1rmf	3/61	1.8	0.01	16.7
	1tet	2/61	2.6	−0.15	16.7
	1ucb	1/61	4.3	0.33	16.7
	1vfa	1/61	2.5	0.20	16.7
	1vge	3/61	2.1	0.03	16.7
	1yuh	22/61	0.6	−0.02	16.7
	2cgr	1/60	2.5	0.17	16.7
	2fb4	19/61	0.4	−0.12	0.0
	2fbj	53/61	−0.9	0.12	16.7
	2gfb	1/61	2.9	0.28	16.7
	3hfl	12/61	1.0	0.20	16.7
	3hfm	61/61	−4.3	−0.16	0.0
	6fab	7/61	1.4	0.02	0.0
	7fab	23/61	0.3	0.01	0.0
	8fab	1/61	3.1	0.04	16.7

lmds	1b0nB	1/498	3.9	0.05	10.2
	1bba	496/501	−2.1	0.03	18.0
	1ctf	32/498	1.6	0.03	14.3
	1dtk	206/216	−1.7	0.04	14.3
	1fc2	247/501	0.0	0.05	16.0
	1igd	342/501	−0.4	0.14	10.0
	1shfA	223/438	0.1	−0.01	4.7
	2cro	2/501	2.3	0.22	14.0
	2ovo	110/348	0.4	0.05	11.8
	4pti	26/344	1.5	0.02	14.7
	smd3	1/501	3.5	0.01	10.0

^*∗*^Native rank = (rank of native structure with given PDB ID)/(total number of decoys); a rank of 1 is optimal and means the calculated energy of the native structure is lower than that of all its decoys; *r* = correlation coefficient; FE = fractional enrichment.

**Table 4:** Relative performance among twelve atomic four-body statistical potentials.

Alphabet size	4	4	4	4	8	8	8	8	20	20	20	20
Cutoff (Å)	4.8	8	12	None	4.8	8	12	None	4.8	8	12	None

^*∗*^Native rank	42	48	55	61	23	29	27	25	33	41	34	33
	(4)	(3)	(2)	(1)	(12)	(9)	(10)	(11)	(7)	(5)	(6)	(7)

^*∗*^ *z*-score	25	9	18	37	1	4	1	1	15	21	2	11
	(2)	(7)	(4)	(1)	(10)	(8)	(10)	(10)	(5)	(3)	(9)	(6)

^*∗*^ *r*	10	9	6	24	4	6	7	9	5	28	21	16
	(5)	(6)	(9)	(2)	(12)	(9)	(8)	(6)	(11)	(1)	(3)	(4)

^*∗*^FE	38	46	49	47	23	34	32	38	37	56	43	42
	(7)	(4)	(2)	(3)	(12)	(10)	(11)	(7)	(9)	(1)	(5)	(6)

Average of ranks	4.5	5	4.25	1.75	11.5	9	9.75	8.5	8	2.5	5.75	5.75
Overall ranking	4	5	3	1	12	10	11	9	8	2	6	6

^*∗*^Numbers above parentheses in each row reflect how many decoy sets (out of 145) for which the given potential matches the best performance value achieved among all 12 potentials tested; numbers in parentheses are the rankings of the counts in that row; *r* = correlation coefficient; FE = fractional enrichment.

**Table 5:** Relative performance among the four-body potential derived using a 4-letter alphabet and 12 Å cutoff as parameters and twelve other state-of-the-art methods.

	^*∗*^Native rank	^*∗*^ *z*-score	^*∗*^ *r*	^*∗*^FE	Average of ranks	Overall ranking
4 letters/12 Å cutoff	47 (5)	17 (3)	8 (5)	27 (5)	4.5	3
Summa et al. [3]	97 (1)	44 (1)	7 (6)	19 (8)	4	2
United-atom vdW (AMBER) [19]	25 (12)	0 (12)	4 (8)	10 (12)	11	12
Coulombic (AMBER) [19]	33 (8)	8 (6)	2 (11)	18 (9)	8.5	10
United-atom vdW + coulombic (AMBER) [19]	26 (11)	4 (9)	4 (8)	11 (11)	9.75	11
United-atom vdW (CHARM19) [20]	31 (10)	9 (4)	4 (8)	36 (3)	6.25	7
Coulombic (CHARM19) [20]	76 (2)	22 (2)	5 (7)	22 (7)	4.5	3
Δ*E* (Delarue and Koehl) [21]	70 (3)	7 (7)	0 (13)	17 (10)	8.25	9
Δ*G*^env^ (Koehl and Delarue) [22]	33 (8)	0 (12)	32 (2)	26 (6)	7	8
Δ*E*^solv^ (Delarue and Koehl) [21]	20 (13)	1 (11)	1 (12)	9 (13)	12.25	13
RAPDF [23]	53 (4)	3 (10)	28 (3)	36 (3)	5	5
DFIRE [24]	38 (7)	9 (4)	33 (1)	51 (1)	3.25	1
Fogolari et al. [18]	40 (6)	5 (8)	15 (4)	39 (2)	5	5

^*∗*^Numbers not in parentheses in each column reflect how many decoy sets (out of 129) for which the given method matches the best performance value achieved among all 13 methods tested; numbers in parentheses are the rankings of the counts in that column; *r* = correlation coefficient; FE = fractional enrichment.

**Table 6:** Overall rankings by separately comparing each four-body potential with twelve other methods.

Alphabet size	4	4	^*∗*^4	4	8	8	8	8	20	20	20	20
Cutoff (Å)	4.8	8	^*∗*^12	None	4.8	8	12	None	4.8	8	12	None
Four-body potential	10	4	3	3	12	11	12	11	11	9	10	11
Summa et al. [3]	2	2	2	2	2	2	2	2	2	2	2	2
United-atom vdW (AMBER) [19]	12	12	12	12	11	12	11	12	12	12	12	12
Coulombic (AMBER) [19]	9	10	10	9	9	9	9	9	9	9	9	9
United-atom vdW + coulombic (AMBER) [19]	11	11	11	11	10	10	10	10	10	11	11	10
United-atom vdW (CHARM19) [20]	6	7	7	7	6	6	6	6	6	6	6	6
Coulombic (CHARM19) [20]	3	3	3	3	3	3	3	2	3	2	3	2
Δ*E* (Delarue and Koehl) [21]	8	9	9	10	8	8	8	8	8	8	8	8
Δ*G*^env^ (Koehl and Delarue) [22]	7	8	8	8	7	7	7	7	7	7	7	7
Δ*E*^solv^ (Delarue and Koehl) [21]	13	13	13	13	12	13	13	13	13	13	13	13
RAPDF [23]	5	5	5	5	5	5	5	5	5	5	5	5
DFIRE [24]	1	1	1	1	1	1	1	1	1	1	1	1
Fogolari et al. [18]	4	5	5	5	4	4	4	4	4	4	4	4

^*∗*^Note that the overall rankings in this column correspond to those in the final column of Table 5; the remaining columns in this table were obtained by repeating the data analyses that generated Table 5 with respect to each of the other 11 four-body potentials.

**Table 7:** Summary data for the 1417 PDB coordinate files.

Atom types	Count	Proportion
C (carbon)	3612988	0.633193
N (nitrogen)	969253	0.169866
O (oxygen)	1088410	0.190749
S (sulfur)	28502	0.004995
M (all metals)	2529	0.000443
X (all other nonmetals)	4299	0.000754

Total atom count	5705981	

Total tetrahedron count	36406467	

**Table 8:** All-atom four-body statistical potential derived using a 6-letter alphabet and a 12 Å cutoff.

Quad	Count	*f*_*ijkl*_	*p*_*ijkl*_	*s*_*ijkl*_
CCCC	4107297	0.112818	0.160748	−0.15377
CCCM	1924	5.28*E* − 05	0.00045	−0.93026
CCCN	4142684	0.11379	0.172495	−0.18067
CCCO	6462239	0.177503	0.193701	−0.03793
CCCS	297980	0.008185	0.005072	0.207795
CCCX	2996	8.23*E* − 05	0.000765	−0.96834
CCMM	157	4.31*E* − 06	4.73*E* − 07	0.96026
CCMN	3758	0.000103	0.000362	−0.5452
CCMO	6511	0.000179	0.000407	−0.35687
CCMS	2320	6.37*E* − 05	1.07*E* − 05	0.776892
CCMX	15	4.12*E* − 07	1.61*E* − 06	−0.591
CCNN	1871781	0.051413	0.069412	−0.13036
CCNO	8544461	0.234696	0.155892	0.177683
CCNS	128008	0.003516	0.004082	−0.06485
CCNX	2159	5.93*E* − 05	0.000616	−1.01632
CCOO	3686844	0.101269	0.087528	0.063328
CCOS	205846	0.005654	0.004584	0.091103
CCOX	4995	0.000137	0.000691	−0.7024
CCSS	15467	0.000425	6.00*E* − 05	0.849914
CCSX	148	4.07*E* − 06	1.81*E* − 05	−0.64875
CCXX	161	4.42*E* − 06	1.37*E* − 06	0.510349
CMMM	29	7.97*E* − 07	2.21*E* − 10	3.557768
CMMN	164	4.50*E* − 06	2.54*E* − 07	1.249604
CMMO	293	8.05*E* − 06	2.85*E* − 07	1.451272
CMMS	665	1.83*E* − 05	7.46*E* − 09	3.389144
CMMX	1	2.75*E* − 08	1.12*E* − 09	1.38783
CMNN	2643	7.26*E* − 05	9.72*E* − 05	−0.12663
CMNO	7243	0.000199	0.000218	−0.0402
CMNS	2610	7.17*E* − 05	5.72*E* − 06	1.098444
CMNX	30	8.24*E* − 07	8.62*E* − 07	−0.01957
CMOO	9551	0.000262	0.000123	0.33061
CMOS	1041	2.86*E* − 05	6.42*E* − 06	0.648899
CMOX	77	2.12*E* − 06	9.68*E* − 07	0.339447
CMSS	2052	5.64*E* − 05	8.40*E* − 08	2.826573
CMSX	13	3.57*E* − 07	2.53*E* − 08	1.148817
CMXX	6	1.65*E* − 07	1.91*E* − 09	1.935563
CNNN	122810	0.003373	0.012414	−0.56586
CNNO	2117811	0.058171	0.041821	0.143315
CNNS	16884	0.000464	0.001095	−0.37318
CNNX	631	1.73*E* − 05	0.000165	−0.97912
CNOO	2981894	0.081906	0.046962	0.241565
CNOS	99630	0.002737	0.00246	0.04635
CNOX	2400	6.59*E* − 05	0.000371	−0.75032
CNSS	4318	0.000119	3.22*E* − 05	0.56619
CNSX	38	1.04*E* − 06	9.71*E* − 06	−0.96883
CNXX	68	1.87*E* − 06	7.33*E* − 07	0.406432
COOO	683049	0.018762	0.017579	0.028291
COOS	38976	0.001071	0.001381	−0.11057
COOX	24064	0.000661	0.000208	0.50151
COSS	4524	0.000124	3.62*E* − 05	0.536074
COSX	64	1.76*E* − 06	1.09*E* − 05	−0.79279
COXX	84	2.31*E* − 06	8.23*E* − 07	0.447847
CSSS	320	8.79*E* − 06	3.16*E* − 07	1.44474
CSSX	5	1.37*E* − 07	1.43*E* − 07	−0.01705
CSXX	4	1.10*E* − 07	2.15*E* − 08	0.707545
CXXX	12	3.30*E* − 07	1.08*E* − 09	2.483295
MMMM	83	2.28*E* − 06	3.86*E* − 14	7.771426
MMMN	42	1.15*E* − 06	5.92*E* − 11	4.290048
MMMO	31	8.51*E* − 07	6.64*E* − 11	4.107805
MMMS	379	1.04*E* − 05	1.74*E* − 12	6.777
MMMX	0	0	2.62*E* − 13	—
MMNN	85	2.33*E* − 06	3.40*E* − 08	1.836638
MMNO	113	3.10*E* − 06	7.64*E* − 08	1.608913
MMNS	364	1.00*E* − 05	2.00*E* − 09	3.698853
MMNX	0	0	3.02*E* − 10	—
MMOO	320	8.79*E* − 06	4.29*E* − 08	2.311659
MMOS	104	2.86*E* − 06	2.25*E* − 09	3.104429
MMOX	3	8.24*E* − 08	3.39*E* − 10	2.386025
MMSS	254	6.98*E* − 06	2.94*E* − 11	5.375177
MMSX	2	5.49*E* − 08	8.87*E* − 12	3.791851
MMXX	0	0	6.69*E* − 13	—
MNNN	1048	2.88*E* − 05	8.69*E* − 06	0.520184
MNNO	1323	3.63*E* − 05	2.93*E* − 05	0.093906
MNNS	562	1.54*E* − 05	7.67*E* − 07	1.303999
MNNX	6	1.65*E* − 07	1.16*E* − 07	0.153922
MNOO	4193	0.000115	3.29*E* − 05	0.544515
MNOS	352	9.67*E* − 06	1.72*E* − 06	0.74942
MNOX	31	8.51*E* − 07	2.60*E* − 07	0.515747
MNSS	793	2.18*E* − 05	2.25*E* − 08	2.985098
MNSX	5	1.37*E* − 07	6.80*E* − 09	1.305273
MNXX	9	2.47*E* − 07	5.13*E* − 10	2.683083
MOOO	5790	0.000159	1.23*E* − 05	1.111435
MOOS	167	4.59*E* − 06	9.67*E* − 07	0.676269
MOOX	171	4.70*E* − 06	1.46*E* − 07	1.508056
MOSS	211	5.80*E* − 06	2.53*E* − 08	2.359752
MOSX	4	1.10*E* − 07	7.64*E* − 09	1.158007
MOXX	55	1.51*E* − 06	5.76*E* − 10	3.418848
MSSS	62	1.70*E* − 06	2.21*E* − 10	3.8869
MSSX	2	5.49*E* − 08	1.00*E* − 10	2.739925
MSXX	0	0	1.51*E* − 11	—
MXXX	16	4.39*E* − 07	7.58*E* − 13	5.763152
NNNN	5639	0.000155	0.000833	−0.7304
NNNO	60175	0.001653	0.00374	−0.35461
NNNS	538	1.48*E* − 05	9.79*E* − 05	−0.82132
NNNX	39	1.07*E* − 06	1.48*E* − 05	−1.13953
NNOO	384854	0.010571	0.006299	0.224828
NNOS	6209	0.000171	0.00033	−0.28656
NNOX	354	9.72*E* − 06	4.98*E* − 05	−0.70907
NNSS	319	8.76*E* − 06	4.32*E* − 06	0.307157
NNSX	6	1.65*E* − 07	1.30*E* − 06	−0.898
NNXX	7	1.92*E* − 07	9.83*E* − 08	0.29148
NOOO	227156	0.006239	0.004716	0.121592
NOOS	11871	0.000326	0.00037	−0.05545
NOOX	3214	8.83*E* − 05	5.59*E* − 05	0.198618
NOSS	951	2.61*E* − 05	9.70*E* − 06	0.430162
NOSX	13	3.57*E* − 07	2.93*E* − 06	−0.9136
NOXX	66	1.81*E* − 06	2.21*E* − 07	0.914541
NSSS	35	9.61*E* − 07	8.47*E* − 08	1.055088
NSSX	0	0	3.83*E* − 08	—
NSXX	0	0	5.78*E* − 09	—
NXXX	3	8.24*E* − 08	2.91*E* − 10	2.452665
OOOO	61473	0.001689	0.001324	0.105657
OOOS	5019	0.000138	0.000139	−0.00255
OOOX	9614	0.000264	2.09*E* − 05	1.101242
OOSS	331	9.09*E* − 06	5.45*E* − 06	0.222484
OOSX	45	1.24*E* − 06	1.64*E* − 06	−0.12365
OOXX	144	3.96*E* − 06	1.24*E* − 07	1.504034
OSSS	38	1.04*E* − 06	9.51*E* − 08	1.040448
OSSX	3	8.24*E* − 08	4.30*E* − 08	0.282172
OSXX	0	0	6.49*E* − 09	—
OXXX	5	1.37*E* − 07	3.26*E* − 10	2.624158
SSSS	11	3.02*E* − 07	6.23*E* − 10	2.686034
SSSX	0	0	3.76*E* − 10	—
SSXX	0	0	8.50*E* − 11	—
SXXX	0	0	8.55*E* − 12	—
XXXX	0	0	3.22*E* − 13	—

**Table 9:** PDB accession codes for 140 HIV-1 protease-inhibitor complexes.

Twenty-five complexes culled from Jenwitheesuk and Samudrala [25]												
1gno	1hbv	1hef	1heg	1hih	1hiv	1hps	1hpv	1hte	1htf	1htg	1hvi	1hvj
1hvk	1hvl	1hvr	1hvs	1pro	1sbg	2upj	4hvp	4phv	5hvp	8hvp	9hvp	

An additional 115 complexes obtained from the Binding MOAD database [26, 27]												
1a30	1a8g	1a8k	1a94	1a9m	1ajv	1b6j	1b6k	1b6m	1b6p	1c70	1d4h	1d4i
1d4k	1d4l	1daz	1dmp	1dw6	1ebk	1ebw	1eby	1ebz	1ec1	1ec2	1ec3	1fej
1ff0	1fff	1ffi	1fg8	1fgc	1fqx	1g2k	1g35	1gnm	1gnn	1hpo	1hsg	1hwr
1hxb	1iiq	1izh	1k1t	1k1u	1k2b	1k2c	1k6p	1k6v	1lzq	1m0b	1met	1mrx
1msn	1mtr	1nh0	1odw	1ody	1ohr	1qbr	1qbs	1qbt	1qbu	1sdu	1t7k	1tcx
1vij	1vik	1z1h	1zj7	1zlf	1zp8	1zpa	2aod	2aog	2aqu	2avm	2avo	2bpv
2bqv	2cem	2cen	2f3k	2f80	2fgu	2idw	2ien	2ieo	2nxd	2nxl	2nxm	2o4s
2p3b	2psu	2psv	2pym	2pyn	2q54	2q55	2q5k	2q63	2qd6	2qd8	2qi1	2qi4
2qi7	2r38	2wkz	2wl0	3bgb	3cyw	3cyx	3d1x	3d1y	3d1z	3d20		
