# Corrigendum to “All-Atom Four-Body Knowledge-Based Statistical Potentials to Distinguish Native Protein Structures from Nonnative Folds”

**DOI:** 10.1155/2018/7108272

**Published:** 2018-01-08

**Authors:** Majid Masso

**Affiliations:** School of Systems Biology, George Mason University, 10900 University Blvd. MS 5B3, Manassas, VA 20110, USA

 In the article titled “All-Atom Four-Body Knowledge-Based Statistical Potentials to Distinguish Native Protein Structures from Nonnative Folds” [1], there were errors in the energy values in the last column of Table 8. The corrected table is shown below.

## Figures and Tables

**Table 8:** All-atom four-body statistical potential derived using a 6-letter alphabet and a 12 Å cutoff.

Quad	Count	*f*_*ijkl*_	*p*_*ijkl*_	*s*_*ijkl*_
CCCC	4107297	0.112818	0.160748	0.15377
CCCM	1924	5.28*E* − 05	0.00045	0.93026
CCCN	4142684	0.11379	0.172495	0.18067
CCCO	6462239	0.177503	0.193701	0.03793
CCCS	297980	0.008185	0.005072	−0.207795
CCCX	2996	8.23*E* − 05	0.000765	0.96834
CCMM	157	4.31*E* − 06	4.73*E* − 07	−0.96026
CCMN	3758	0.000103	0.000362	0.5452
CCMO	6511	0.000179	0.000407	0.35687
CCMS	2320	6.37*E* − 05	1.07*E* − 05	−0.776892
CCMX	15	4.12*E* − 07	1.61*E* − 06	0.591
CCNN	1871781	0.051413	0.069412	0.13036
CCNO	8544461	0.234696	0.155892	−0.177683
CCNS	128008	0.003516	0.004082	0.06485
CCNX	2159	5.93*E* − 05	0.000616	1.01632
CCOO	3686844	0.101269	0.087528	−0.063328
CCOS	205846	0.005654	0.004584	−0.091103
CCOX	4995	0.000137	0.000691	0.7024
CCSS	15467	0.000425	6.00*E* − 05	−0.849914
CCSX	148	4.07*E* − 06	1.81*E* − 05	0.64875
CCXX	161	4.42*E* − 06	1.37*E* − 06	−0.510349
CMMM	29	7.97*E* − 07	2.21*E* − 10	−3.557768
CMMN	164	4.50*E* − 06	2.54*E* − 07	−1.249604
CMMO	293	8.05*E* − 06	2.85*E* − 07	−1.451272
CMMS	665	1.83*E* − 05	7.46*E* − 09	−3.389144
CMMX	1	2.75*E* − 08	1.12*E* − 09	−1.38783
CMNN	2643	7.26*E* − 05	9.72*E* − 05	0.12663
CMNO	7243	0.000199	0.000218	0.0402
CMNS	2610	7.17*E* − 05	5.72*E* − 06	−1.098444
CMNX	30	8.24*E* − 07	8.62*E* − 07	0.01957
CMOO	9551	0.000262	0.000123	−0.33061
CMOS	1041	2.86*E* − 05	6.42*E* − 06	−0.648899
CMOX	77	2.12*E* − 06	9.68*E* − 07	−0.339447
CMSS	2052	5.64*E* − 05	8.40*E* − 08	−2.826573
CMSX	13	3.57*E* − 07	2.53*E* − 08	−1.148817
CMXX	6	1.65*E* − 07	1.91*E* − 09	−1.935563
CNNN	122810	0.003373	0.012414	0.56586
CNNO	2117811	0.058171	0.041821	−0.143315
CNNS	16884	0.000464	0.001095	0.37318
CNNX	631	1.73*E* − 05	0.000165	0.97912
CNOO	2981894	0.081906	0.046962	−0.241565
CNOS	99630	0.002737	0.00246	−0.04635
CNOX	2400	6.59*E* − 05	0.000371	0.75032
CNSS	4318	0.000119	3.22*E* − 05	−0.56619
CNSX	38	1.04*E* − 06	9.71*E* − 06	0.96883
CNXX	68	1.87*E* − 06	7.33*E* − 07	−0.406432
COOO	683049	0.018762	0.017579	−0.028291
COOS	38976	0.001071	0.001381	0.11057
COOX	24064	0.000661	0.000208	−0.50151
COSS	4524	0.000124	3.62*E* − 05	−0.536074
COSX	64	1.76*E* − 06	1.09*E* − 05	0.79279
COXX	84	2.31*E* − 06	8.23*E* − 07	−0.447847
CSSS	320	8.79*E* − 06	3.16*E* − 07	−1.44474
CSSX	5	1.37*E* − 07	1.43*E* − 07	0.01705
CSXX	4	1.10*E* − 07	2.15*E* − 08	−0.707545
CXXX	12	3.30*E* − 07	1.08*E* − 09	−2.483295
MMMM	83	2.28*E* − 06	3.86*E* − 14	−7.771426
MMMN	42	1.15*E* − 06	5.92*E* − 11	−4.290048
MMMO	31	8.51*E* − 07	6.64*E* − 11	−4.107805
MMMS	379	1.04*E* − 05	1.74*E* − 12	−6.777
MMMX	0	0	2.62*E* − 13	-* *-
MMNN	85	2.33*E* − 06	3.40*E* − 08	−1.836638
MMNO	113	3.10*E* − 06	7.64*E* − 08	−1.608913
MMNS	364	1.00*E* − 05	2.00*E* − 09	−3.698853
MMNX	0	0	3.02*E* − 10	-* *-
MMOO	320	8.79*E* − 06	4.29*E* − 08	−2.311659
MMOS	104	2.86*E* − 06	2.25*E* − 09	−3.104429
MMOX	3	8.24*E* − 08	3.39*E* − 10	−2.386025
MMSS	254	6.98*E* − 06	2.94*E* − 11	−5.375177
MMSX	2	5.49*E* − 08	8.87*E* − 12	−3.791851
MMXX	0	0	6.69*E* − 13	-* *-
MNNN	1048	2.88*E* − 05	8.69*E* − 06	−0.520184
MNNO	1323	3.63*E* − 05	2.93*E* − 05	−0.093906
MNNS	562	1.54*E* − 05	7.67*E* − 07	−1.303999
MNNX	6	1.65*E* − 07	1.16*E* − 07	−0.153922
MNOO	4193	0.000115	3.29*E* − 05	−0.544515
MNOS	352	9.67*E* − 06	1.72*E* − 06	−0.74942
MNOX	31	8.51*E* − 07	2.60*E* − 07	−0.515747
MNSS	793	2.18*E* − 05	2.25*E* − 08	−2.985098
MNSX	5	1.37*E* − 07	6.80*E* − 09	−1.305273
MNXX	9	2.47*E* − 07	5.13*E* − 10	−2.683083
MOOO	5790	0.000159	1.23*E* − 05	−1.111435
MOOS	167	4.59*E* − 06	9.67*E* − 07	−0.676269
MOOX	171	4.70*E* − 06	1.46*E* − 07	−1.508056
MOSS	211	5.80*E* − 06	2.53*E* − 08	−2.359752
MOSX	4	1.10*E* − 07	7.64*E* − 09	−1.158007
MOXX	55	1.51*E* − 06	5.76*E* − 10	−3.418848
MSSS	62	1.70*E* − 06	2.21*E* − 10	−3.8869
MSSX	2	5.49*E* − 08	1.00*E* − 10	−2.739925
MSXX	0	0	1.51*E* − 11	-* *-
MXXX	16	4.39*E* − 07	7.58*E* − 13	−5.763152
NNNN	5639	0.000155	0.000833	0.7304
NNNO	60175	0.001653	0.00374	0.35461
NNNS	538	1.48*E* − 05	9.79*E* − 05	0.82132
NNNX	39	1.07*E* − 06	1.48*E* − 05	1.13953
NNOO	384854	0.010571	0.006299	−0.224828
NNOS	6209	0.000171	0.00033	0.28656
NNOX	354	9.72*E* − 06	4.98*E* − 05	0.70907
NNSS	319	8.76*E* − 06	4.32*E* − 06	−0.307157
NNSX	6	1.65*E* − 07	1.30*E* − 06	0.898
NNXX	7	1.92*E* − 07	9.83*E* − 08	−0.29148
NOOO	227156	0.006239	0.004716	−0.121592
NOOS	11871	0.000326	0.00037	0.05545
NOOX	3214	8.83*E* − 05	5.59*E* − 05	−0.198618
NOSS	951	2.61*E* − 05	9.70*E* − 06	−0.430162
NOSX	13	3.57*E* − 07	2.93*E* − 06	0.9136
NOXX	66	1.81*E* − 06	2.21*E* − 07	−0.914541
NSSS	35	9.61*E* − 07	8.47*E* − 08	−1.055088
NSSX	0	0	3.83*E* − 08	-* *-
NSXX	0	0	5.78*E* − 09	-* *-
NXXX	3	8.24*E* − 08	2.91*E* − 10	−2.452665
OOOO	61473	0.001689	0.001324	−0.105657
OOOS	5019	0.000138	0.000139	0.00255
OOOX	9614	0.000264	2.09*E* − 05	−1.101242
OOSS	331	9.09*E* − 06	5.45*E* − 06	−0.222484
OOSX	45	1.24*E* − 06	1.64*E* − 06	0.12365
OOXX	144	3.96*E* − 06	1.24*E* − 07	−1.504034
OSSS	38	1.04*E* − 06	9.51*E* − 08	−1.040448
OSSX	3	8.24*E* − 08	4.30*E* − 08	−0.282172
OSXX	0	0	6.49*E* − 09	-* *-
OXXX	5	1.37*E* − 07	3.26*E* − 10	−2.624158
SSSS	11	3.02*E* − 07	6.23*E* − 10	−2.686034
SSSX	0	0	3.76*E* − 10	-* *-
SSXX	0	0	8.50*E* − 11	-* *-
SXXX	0	0	8.55*E* − 12	-* *-
XXXX	0	0	3.22*E* − 13	-* *-
